# DNA repair: Location, location, location

**DOI:** 10.18632/oncotarget.4840

**Published:** 2015-07-11

**Authors:** Marjolein van Sluis, Brian McStay

**Affiliations:** Centre for Chromosome Biology, School of Natural Sciences, National University of Ireland Galway, Ireland

**Keywords:** Chromosome Section

DNA double strand breaks (DSBs) are the most dangerous form of DNA damage and need to be repaired efficiently in order to maintain genomic stability. The kinase ATM becomes activated upon recognition of DSBs and transduces this into cell cycle checkpoint activation and DNA repair. There are two main pathways by which DSBs can be repaired: Non-homologous end joining (NHEJ) and homologous recombination (HR). In NHEJ, the broken ends are minimally processed and after sequence-independent alignment, ligated back together often resulting in point mutations, small deletions and insertions. In the HR pathway, extensive DNA end resection produces single-stranded DNA, which subsequently invades a homologous DNA duplex that in turn templates new DNA synthesis and repair. Usually sister chromatids provide the repair template, thus HR is normally considered to be restricted to S and G2 phases of the cell cycle. The NHEJ versus HR pathway choice is also influenced by the genomic and nuclear location of DSBs [1]. As nucleoli are the largest and most visible functional domains of the nucleus and contain the most actively transcribed genes in all eukaryotes, they offer an opportunity to study temporal and regional influences on repair pathway choice.

Nucleoli, the sites of ribosome biogenesis, form around arrays of ribosomal gene (rDNA) repeats that are transcribed by the dedicated RNA polymerase I (pol I) transcription machinery. In humans, rDNA arrays, termed nucleolar organiser regions (NORs), are positioned on the short arms of the five acrocentric chromosomes. While rDNA repeats are localized within the nucleolar interior, NOR distal sequences, termed distal junction (DJ), are embedded in peri-nucleolar heterochromatin [2].

In recent years, several groups have studied DSBs within the nucleolus using γ-irradiation and micro-irradiation. These studies have yielded conflicting results, ranging from complete ATM-dependent inhibition of nucleolar transcription to no observable effect on nucleolar function [3, 4]. In order to more precisely control the location of DSBs, we have recently exploited the homing endonuclease I-PpoI from *Physarum*, which has a recognition sequence once in each of the ~300 rDNA repeats and up to 13 sites elsewhere in the genome [5].

Introduction of I-PpoI into human cells causes ATM-dependent inhibition of pol I transcription followed by a reorganization of the nucleolar structure to form so-called caps at the nucleolar periphery. Using CRISPR/Cas9, we revealed that DSB introduced across the rDNA repeat elicit the same response. This movement of damaged rDNA to the nucleolar periphery facilitates repair. In contrast, DSBs introduced into DJ sequences do not exhibit this response. Nucleolar caps containing rDNA DSBs are highly enriched in activated ATM and components of the HR machinery. No enrichment of NHEJ factors is observed. These results reveal how genomic location and nuclear position influence repair choice [5].

Surprisingly, HR factors could be observed in the majority of nucleolar caps, raising the possibility of repair by HR in G1 cells, where sister chromatids are absent. By exploiting the FUCCI system (fluorescence ubiquitination cell cycle indicator [6]), HR factors can be readily detected at nucleolar caps in G1 cells. As further evidence that repair can occur in G1 cells, damage-induced DNA synthesis can also be observed within G1 nucleolar caps [5].

These results strongly suggest that within repetitive rDNA arrays, DSBs can be repaired by HR independent of the cell cycle, probably templated by undamaged rDNA repeats *in cis*. HR templated *in cis* need not involve cross-overs with loss or gain of sequence. Indeed, previous evidence indicates that the Blooms helicase (BLM)-dependent branch migration pathway promotes non-crossover HR within rDNA arrays [7].

Future work will be needed to determine if repair is completed within G1 cells, and to assess the role of chromosomal context in maintaining the genomic stability of rDNA arrays.

Apart from advancing our understanding of the nucleolar response to DSBs, our recent work also presents a number of technical advances that may be of interest to other workers in the DNA damage field. Transfection of cells with synthetic mRNA is efficient in a wide variety of cell types, independent of cell cycle, and importantly, does not itself induce a DNA damage response. To our knowledge, this is the first use of the CRISPR/Cas9 system to probe DSB responses at specific chromosomal sites. Moreover, by using targeted introduction of DSBs in combination with the following techniques; antibody staining, FUCCI, EdU incorporation and FISH, repair processes at specific chromosomal sites can be studied *in vivo* [5].
